# Barriers and facilitators to advance care planning for people with intellectual disabilities: a cross-sectional survey study of professional caregiver perspectives

**DOI:** 10.1186/s12939-025-02747-1

**Published:** 2025-12-27

**Authors:** Elisabeth Lucia Zeilinger, Lena Simeoni, Theresa Wagner, Tamina-Laetitia Vielgrader, Amelie Fuchs, Tobias Fragner, Igor Grabovac, Eva Katharina Masel, Matthias Unseld

**Affiliations:** 1 Department of Clinical and Health Psychology, Faculty of Psychology, University of Vienna, Liebiggasse 5, Vienna, A-1010 Austria; 2 Department of Clinical Research SBG, Academy for Ageing Research, Haus der Barmherzigkeit, Vienna, Austria; 3 Division of Health Psychology, Faculty of Psychology, Karl Landsteiner University of Health Sciences, Krems, Austria; 4 Division of Palliative Medicine, Department of Medicine I, Medical University of Vienna, Vienna, Austria; 5 Institute for Ethics and Law in Medicine, University of Vienna, Vienna, Austria; 6 Vienna Doctoral School in Cognition, Behavior and Neuroscience, University of Vienna, Vienna, Austria; 7 Department of Social and Preventive Medicine, Centre for Public Health, Medical University of Vienna, Vienna, Austria

**Keywords:** Advance care planning, End-of-life care, Health equity, Inclusive healthcare, Intellectual disabilities, Learning disabilities, Palliative care, Person-centred care

## Abstract

**Background:**

Advance care planning (ACP) is a critical process for ensuring person-centred end-of-life care, yet it remains underutilized among people with intellectual disabilities (ID). Understanding caregivers’ perspectives is essential to identify barriers and facilitators to ACP implementation and improve practice. This study aimed to examine how professional caregivers in Austria perceive and experience ACP for people with ID, including its current use, barriers, facilitators, and strategies to improve uptake.

**Methods:**

A cross-sectional survey was conducted using a structured online form comprising multiple-choice and open-ended questions. Data were collected from 125 professional caregivers across Austria who were primary caregivers of at least one adult with ID and proficient in German. Quantitative data were analysed descriptively, while qualitative responses to open-ended questions were subjected to content analysis.

**Results:**

A total of 33.6% of caregivers reported engaging in ACP discussions, with considerable barriers including cognitive and communicative challenges, emotional discomfort, and structural constraints. Facilitators included person-centred communication, interdisciplinary collaboration, and targeted training. Notably, 83.2% of caregivers expressed interest in ACP training.

**Conclusions:**

ACP is rarely practiced in the care of people with ID in Austria. However, caregivers identified clear pathways to improve implementation, particularly through training, use of tailored communication methods, and systemic support within care institutions. Promoting inclusive ACP practices is essential to uphold the autonomy and health equity of people with ID, ensuring their voices are heard in decisions about their future and end-of-life care.

**Supplementary Information:**

The online version contains supplementary material available at 10.1186/s12939-025-02747-1.

## Plain Language summary

People with intellectual disabilities often do not get the chance to talk about their wishes for future care and end-of life decisions. Advance care planning (ACP) means talking about what kind of care a person wants later in life, especially if they get very sick. It helps make sure their wishes are known and respected. In this study, we asked 125 professional caregivers in Austria about their experiences with ACP. We wanted to know how often they talk about future care, what makes it easy or hard, and how it can become better. Only one in three caregivers said they had talked about ACP with the person they care for. Many said it was difficult because of communication problems, not enough time, or not knowing how to do it. But most caregivers said they want more training and believe ACP is important. The study shows that people with intellectual disabilities need more support to take part in ACP. Professional caregivers need training, more time, and tools like pictures or simple language to help with these talks. This can help people with intellectual disabilities be more involved in decisions about their care and future.

**Supplementary Information:**

The online version contains supplementary material available at 10.1186/s12939-025-02747-1.

## Background

End-of-life discussions and decisions are a critical part of healthcare, necessitating a person-centred approach that respects individuals’ autonomy, preferences, and rights. For individuals with intellectual disabilities (ID), these conversations are particularly crucial due to their historical and ongoing exclusion from equitable healthcare and decision-making processes [1].

ID are characterised by significant limitations in intellectual functioning (IQ < 70) and adaptive behaviour, incorporating social, conceptual, and practical skills, and emerging during the individual’s developmental phase [2]. As people with ID age, they often experience complex health needs, including a higher prevalence of chronic conditions and multimorbidity [3–5]. From a public health perspective, these needs are further compounded by fragmented care systems, limited access to coordinated services, a lack of inclusive planning, lack of information, poor diagnostic recognition and a delayed integration of end-of-life care into the overall healthcare process [6–10].

Advance care planning (ACP) represents a dynamic, ongoing process of communication among individuals, healthcare providers, and often families or legal representatives about preferences for future healthcare and treatment. It is a broad and flexible concept that aims to document and respect an individual’s preferences for future medical care, not only in clinical but also in psychological, social, and spiritual dimensions [11]. Unlike static tools such as living wills or legal guardianship, ACP fosters a values-based, evolving dialogue that anticipates changing health conditions and facilitates shared decision-making before critical situations arise [12–14].

Although individuals should be especially engaged when their health condition deteriorates or as they age, ACP can be implemented at any stage of life [11]. Planning in advance can prevent difficult decisions during emotionally charged or time-limited situations. By stating their preferences and goals before their condition deteriorates, patients can play a more active role in their own end-of-life care [15].

The effectiveness of ACP has been widely demonstrated across various populations, particularly in improving the quality of end-of-life care, reducing unnecessary hospitalizations, and enhancing satisfaction with care among both patients and their families [12, 16, 17]. However, ACP remains underutilized and under-researched in the context of ID [17]. Numerous barriers hinder its implementation: healthcare professionals often avoid proactively assessing care needs or initiating end-of-life conversations with people with ID due to discomfort or fear of emotional resistance from families [1, 18]. Even within palliative care, staff report feeling unprepared to discuss death and dying with individuals who have ID [19]. Ambiguity around who should initiate ACP, alongside a general lack of knowledge and training, further complicates its application in care settings [20].

People with ID are often not involved in their own end-of-life care, as they are seen as lacking in decision-making capacity, in need of protection, and unable to express choice [1]. A UK-wide survey of intellectual disability support staff found that approximately half of terminally ill people with ID were not informed about their condition, highlighting significant gaps in communication about death and dying within care settings [21]. However, people with ID want to be informed about their current health situation, and they want their family members to support them when making decisions [22, 23]. Furthermore, they want to be listened to by professionals, who should offer them room for asking questions and sharing concerns about their future care and giving accessible information about their options [24]. ACP thus offers a meaningful pathway for affirming their autonomy and enabling supported decision-making [25], aligning with the principle of self-determination, which is closely linked to quality of life and service satisfaction among people with disabilities [26].

Caregivers play a central role in the ACP process for people with ID [27, 28]. In many cases, they act as key facilitators of communication, decision-making, and continuity of care, especially when the person with ID requires support in understanding or expressing preferences. Research has shown that caregiver attitudes, skills, and institutional environments significantly influence whether and how ACP is implemented [19, 23, 27]. Moreover, caregivers often serve as proxies for healthcare professionals in identifying changes in health status and initiating sensitive conversations [19, 27, 28].

In Austria, the implementation of ACP for individuals with ID remains inconsistent and an evaluation is lacking. Legal frameworks such as the Austrian Patient Directive Act and the Adult Protection Law provide avenues for pre-emptive healthcare planning, yet practical applications of ACP in care institutions are limited [29]. Austria’s national disability action plan emphasizes autonomy and individualized support but omits explicit reference to ACP.

From an ethical and rights-based standpoint, it is important to encourage ACP for people with ID. This group has historically been marginalised in medical decision-making processes and has often had to rely on proxies whose decisions may not reflect their true values [28, 30]. A rights-based approach, rooted in the United Nations Convention on the Rights of Persons with Disabilities (UN-CRPD) [31], advocates for inclusive practices that actively engage people with disabilities in decisions affecting their lives, including healthcare and end-of-life decisions.

This study aims to examine how ACP is currently implemented for people with ID in Austria by analysing the perceptions and experiences of primary professional caregivers, who play a pivotal role in either facilitating or impeding such processes. It seeks to identify the main barriers and facilitators of ACP implementation, contributing to broader health equity efforts to ensure that people with ID are included in person-centred care planning processes and supported in expressing their values and preferences.

## Methods

### Study design

This study employed a cross-sectional survey design to examine how professional caregivers in Austria engage with and implement ACP for their clients with ID who they feel particularly responsible for in health-related matters. The online survey combined both quantitative and qualitative methods to capture a comprehensive understanding of the topic. Reporting followed the *Consensus-Based Checklist for Reporting of Survey Studies* (CROSS) [32]. The study received ethical approval from the Ethics Committee of the Medical University of Vienna (No. 1999/2022). All participants gave informed consent before proceeding with the survey. Participants first received study information on the survey’s opening page and provided informed consent by clicking a designated consent button. Contact details were available for those seeking further information before consenting.

### Participants and procedure

The sample consisted of *N* = 125 caregivers working with people with ID across Austria. Participants were recruited between March and August 2023 through a convenience sampling approach, primarily via organisations providing services for people with ID. These organisations were contacted via email and asked to circulate the survey link. Additionally, recruitment materials (e.g., flyers with QR codes) were shared during internal meetings of related social service organizations. We did not implement any measures to prevent people from taking the survey more than once. No remuneration was offered for participation.

Inclusion criteria were: (1) Age ≥ 18 years, (2) being a professional and primary caregiver for a person with ID, (3) sufficient proficiency in German-language to complete the survey. Participation was voluntary and data was collected anonymously without any identifying information.

### Materials

A survey was developed by the research team based on published literature (e.g., [11, 17, 21]) and professional experience. The literature informed the selection of key domains, such as training needs and the presence of chronic illnesses. The multidisciplinary expertise of the research team – comprising clinicians and psychologists with experience in palliative care and ID services – guided the relevance and wording of the survey questions. Thus, the final survey was shaped by both evidence from the literature and the team’s professional experience. The survey was pilot tested with two caregivers known to the research team, who were invited to provide feedback. Based on their input, the survey was refined, primarily through adjusting the wording of the items. The pilot testers did not participate in the final study. The survey for this study comprised three main sections (see Supplementary File 1). Section  “Background” (Sociodemographics) included four items assessing participants’ background characteristics, consisting of two multiple-choice questions and two open-ended questions. Section  “Results” (Training and Educational Needs) contained four multiple-choice items covering prior training experiences and perceived needs for training related to ACP, death, dying, and grief. Section  “Discussion” focused on a specific reference client for whom the caregiver felt particularly responsible in health-related matters. This section included three questions on reference-client characteristics (two multiple-choice items and one open-ended item), followed by four multiple-choice questions on the utilization of future planning and ACP. Finally, participants responded to two open-ended questions addressing reasons for initiating or not initiating ACP with the reference client, and two open-ended questions exploring perceived barriers and facilitators to ACP implementation. The open-ended questions allowed respondents to elaborate in their own words and provided the qualitative data used in this study. Information about the care setting in which respondents worked with their reference clients (e.g., independent living, supported living, institutional care) was not collected in the survey, neither was the frequency or intensity of contact with the reference client (e.g., days per week, hours per day).

### Data analysis

Data were exported from SoSci Survey [33] and initially reviewed for completeness (i.e., required items answered) and plausibility (i.e., values within predefined, realistic ranges such as feasible age values or valid response categories) in Microsoft Excel. Missing data were addressed based on the structure of the online survey. The survey design required respondents to complete key variables before proceeding, resulting in no true missing responses. Non-informative or placeholder responses (e.g., ‘don’t know,’ ‘no response’) were retained as provided by participants, and no imputation was performed. Survey cases that were largely incomplete due to early termination were excluded before analysis (*n* = 14). Descriptive statistical analyses were conducted using the IBM Statistical Package for the Social Sciences (SPSS, v.29.0) to summarize the demographic characteristics of participants and their clients, as well as the distribution of responses concerning ACP practices. To examine whether ACP discussions were associated with clients’ age and the presence of chronic illness, independent samples t-tests and Chi-square tests were conducted. The significance level (α) was set at 5%.

Qualitative responses to four open-ended questions (reasons for using ACP, reasons for not using ACP, barriers to ACP, and facilitators of ACP) were analysed using inductive content analysis within these four predefined domains set by the survey questions. Because these domains were inherent to the question structure, they were not generated through the analysis itself. Within each domain, however, an inductive analytic procedure was applied [34]. The open-ended responses were often brief and not full sentences, reflecting the typical language and concision of written survey comments. Two researchers, one with a medical background and the other with a psychological background, conducted open coding of all responses, identified recurring concepts, and grouped similar codes into preliminary subcategories. The categories were discussed and refined collaboratively until consensus on the final category structure was reached. All coding, category development and naming were conducted using the original German responses. English translations of illustrative quotes and of the final category labels were produced only during manuscript preparation for publication, and were not used in the analysis itself.

## Results

### Participant characteristics

A total of 125 professional caregivers (68.8% women) participated in the study. The average age of the respondents was 41 years (*SD* = 11, range: 21–63 years). The average professional experience in caregiving roles was 14.3 years (*SD* = 9.4), with a range from 1 to 40 years. See Table 1 for sample characteristics.

**Table 1:** Sociodemographic characteristics, training experience, and training needs of caregivers

Variable	*N* (%)
Age	-
** Mean (SD)** = 41 (± 11), **Range** = 21–63	
Gender	
Female	86 (68.8%)
Male	38 (30.4%)
No response	1 (0.8%)
Highest Completed Education	
University degree / tertiary education	58 (46.6%)
Upper secondary education	29 (23.2%)
Lower or intermediate secondary education	22 (17.6%)
Vocational school / apprenticeship training	9 (7.2%)
Other / not specified	7 (5.4%)
Work experience in intellectual disabilities	-
** Mean (SD)** = 14.29 (± 9,37), **Range** = 1–40	
Education/Profession	
Social care worker / Disability support worker	65 (52%)
Educator / Paedagogue	43 (34.4%)
Other / not specified	17 (13.6%)
Received training on ACP	
Yes	6 (4.8%)
No	119 (95.2%)
Interested in training on ACP	
Yes	104 (83.2%)
No	10 (8.0%)
No response	11 (8.8%)
Received training on death, dying, and grief	
Yes	66 (52.8%)
No	59 (47.2%)
Interested in training on death, dying, and grief	
Yes	101 (80.8%)
No	18 (14.4%)
Not specified	6 (4.8%)

Note. *N* = 125. SD = Standard deviation; ACP = Advance care planning

### Training and educational needs

Only 6 participants (4.8%) reported having received any formal training related to ACP. In contrast, 104 participants (83.2%) expressed interest in receiving such training on ACP in the future. Regarding related topics, 66 participants (52.8%) stated that they had previously attended training or events addressing death, dying, and grief in people with ID, and 101 participants (80.8%) indicated a desire for further training on these related topics (see Table 1).

### Utilization of future planning and ACP

For the questions on ACP, caregivers were asked to focus on one specific client they felt particularly responsible for in health-related matters. The mean age of these reference clients was 47.3 years (*SD* = 14.85), with an age range of 17 to 86 years. Just over half (52%) were identified as female and 44% as male. For the remaining 4% no gender was specified in the survey. The caregivers indicated that 34 (27.2%) of the clients had a serious chronic illness (e.g. cancer, heart disease or lung disease); 86 (68.8%) had no such illness and 5 (4%) did not answer this question. See Table 2 for characteristics of reference clients.

**Table 2:** Sociodemographic characteristics of reference clients

Variable	*N* (%)
Chronic Illness^1^	
Yes	34 (27.2%)
No	86 (68.8%)
Don’t know	1 (0.8%)
No response	4 (3.2%)
Age	-
** Mean (SD)** = 47.3 (± 14.85), **Range** = 17–86	
Gender	
Female	65 (52%)
Male	55 (44%)
Don’t know	1 (0.8%)
No response	4 (3.2%)
Health care proxy	
Yes	17 (13.6%)
No	79 (63.2%)
Don’t know	26 (20.8%)
No reponse	3 (2.4%)
Advance directive	
Yes	4 (3.2%)
No	93 (74.4%)
Don’t know	25 (20%)
No response	3 (2.4%)

Note. SD = Standard deviation, ^1^ Chronic illness was assessed as follows: “Does the person have a serious chronic illness, e.g., cancer, heart defect, lung disease?”

In terms of legal planning, only 13.6% of clients had an official health care proxy. A total of 26 caregivers (20.8%) indicated that they did not know whether a health care proxy existed for their client. Only a small proportion of people with ID (3.2%) had a documented advance directive. Similarly, a significant proportion of caregivers (20%) reported uncertainty regarding the existence of such a directive for their client.

Regarding ACP for their reference client, 42 caregivers (33.6%) reported having held at least one ACP discussion with the respective client. A total of 66 caregivers (52.8%) said they had never held an ACP discussion. The remaining 17 caregivers (13.6%) said they did not know whether such a discussion had taken place. These figures were independent of the client having a chronic illness (χ^2^ (1) = 2.47, *p* = 0.116), and independent of the age of the client (*t*(106) = 0.23; *p* = 0.821).

As to people involved in ACP discussions, the caregiver and the client with ID were the most frequently named participants (30.2% and 29.5%, respectively). Family members were involved in 17.8% of cases, and legal representatives in 13.2%. Additional participants included a diverse set of professionals, such as physicians, facility managers, psychologists, and interpreters. Friends were rarely involved (2.3%).

### Reasons for ACP (non-) usage

A total of 87 caregivers responded to open-ended questions about the reasons for using or not using ACP. Four primary categories emerged through content analysis on the reasons for initiating ACP discussions: (1) *Health-related factors*: Common reasons cited involved deteriorating health status or chronic illness of the client, which prompted the initiation of ACP discussions. (2) *Professional responsibilities*: For some respondents, ACP discussions were part of institutional protocols or standard care procedures within their organization. (3) *Autonomy and planning*: The importance of respecting the client’s right to self-determination and planning for the future was also mentioned as a reason to initiate ACP. (4) *Influence of the client’s environment*: In some responses, external factors such as family wishes or recent deaths within the client’s social circle triggered ACP discussions.

**Fig. 1:**
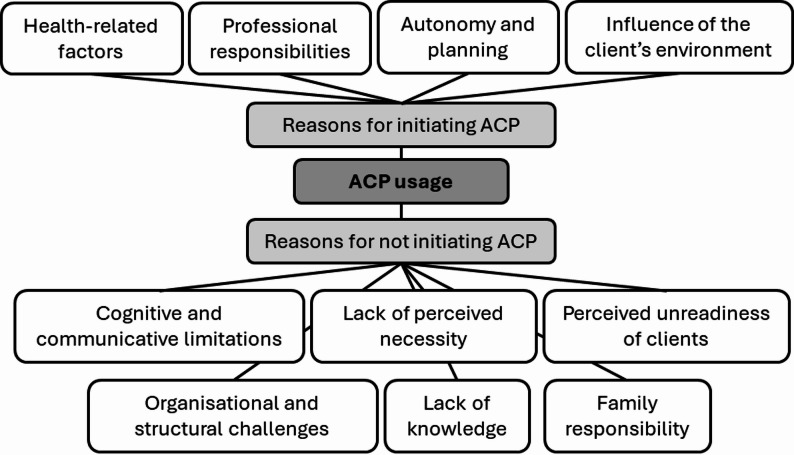
Reasons for initiating or not initiating Advance Care Planning (ACP)

Six main categories were identified for not initiating ACP. (1) *Cognitive and communicative limitations*: One frequently mentioned barrier was the perceived inability of clients to cognitively process or communicate about future care preferences. (2) *Lack of perceived necessity*: Respondents felt there was no immediate need, often due to the client’s young age or stable health status. (3) *Perceived unreadiness of clients*: Within this category, caregivers indicated that they perceived their client as being emotionally uncomfortable or not ready to engage in such discussions. (4) *Organisational and structural challenges*: Other aspects pointed to insufficient institutional planning, staff shortages, and competing priorities. (5) *Lack of knowledge*: In some cases, ACP was not initiated due to caregivers’ unfamiliarity with the concept. (6) *Family responsibility*: Some caregivers deferred ACP responsibility to family members, especially in cases where guardianship arrangements existed. Figure 1 depicts the categories for and against the use of ACP. Table 3 provides selected example responses taken from the open-ended survey items.

**Table 3:** Selected illustrative comments from open-ended survey responses

Domains and Categories	Selected quotes	Participant Number
**Reasons for initiating ACP**		
Health-related factors	Because health is important.	30
	The person’s age	27
	Already having a high need of care and many health issues	13
Professional responsibilities	Because it is part of our admission procedure.	6
	Foresighted planning was a pilot project in the facility.	97
Autonomy and planning	Because the topic is a universally human one and it affects the person’s life now and in the future.	104
	Because she cares about the topic of dying, so that everything is planned in case something happens to her.	31
	Because it’s important to know what the person wants one day.	75
Influence of the client’s environment	So that we can also give the family time to say goodbye and remember that he may die and that he will at some point.	8
	Because she is determined by her mother and thinks highly of what her mother advises her.	7
	Wish of the relatives	59
**Reasons for not initiating ACP**		
Cognitive and communicative limitations	The person does not have the mental capacities to process or understand	33
	The person is communicating non-verbally, her intellectual disability is too severe to provide support with assisted communication.	65
	Difficult to maintain continuous conversation about a topic	70
Lack of perceived necessity	In my opinion there has not been an occasion, he is not old yet, very healthy and so on.	18
	Always though it was still too soon	11
	Other things have a higher priority at the moment.	107
Perceived unreadiness of clients	It can be scary to be questioned about it as a young and healthy individual. It can create the feeling that someone is withholding something about the state of one’s actual health.	19
	The person’s difficulty to deal with the future	3
	The client is not open to talk about these things.	117
Organisational and structural challenges	Discussions like this are not systematically planned in our facility.	4
	Planning ahead is often difficult, as current issues are already coming short.	45
Lack of knowledge	Had never heard of it before	72
	I am unaware of this.	22
	Was not familiar with the concept.	49
Family responsibility	Parents are responsible	34
	She is additionally assisted by her mother. Because of this, I did not see any reason to discuss it yet.	85
**Barriers to ACP**		
Cognitive and communication challenges	Lack of cognitive assessment of the progression of the disease, despite explanation and simplified illustration	22
	Communication is restricted, because the client is non-verbal	12
	Lack of understanding for death, serious illnesses and what that is exactly, understanding when it happens and the necessity to plan ahead in such case.	18
Emotional and individual challenges	I don’t want to worry the person for no reason.	4
	Ideas for the future and fear of death	42
	The person easily refuses everything.	16
Structural barriers and taboos	Financial and time resources. Conversations require a lot of time and empathy, that is impossible in a normal care context.	99
	Communications and working material for these topics is hardly available, or only possible with children’s books.	74
	The facility I work in does not regularly address this. Getting old and dying is still a taboo subject.	35
Challenges in caregiving	I would not feel prepared enough. If I were to do that, I would try to get support from a competent source.	15
	I would need enough time, trust needs to be established, and you need to find the right words.	45
	The person’s wish cannot be clearly recognised.	2
Involvement of family members and legal representatives	The conversation with the family. Because it’s a taboo subject for most people, it’s always a challenge to have this conversation.	8
	Different views between people with intellectual disabilities and their family or legal representatives	87
	Lack of understanding of the importance of this topic among family members and legal representatives	43
**Facilitators of ACP**		
Person-centred communication	More conversations, that slowly lead to this topic, to allow the person to engage with it. Creating a good atmosphere as far as possible.	512
	Use familiar people as examples, for example people with serious illnesses or old people with support needs. Possibly compare photos from the past in which they are young and healthy with current photos. Have people with disabilities talk about their experiences with these people.	270
	Choose the right place and time, postpone if it gets too much, explain to the person that they can say and should say no if they do not want something, use easy-to-understand language, ask if terms are clear.	415
Communication methods	Making use of assisted communication, for example visual representation of life cycle or physical changes in the course of life.	271
	Material to talk about specific topics	498
	Speak in easy-to-understand language so that the person understands	593
Knowledge transfer	Training all employees on the content of palliative care and planning ahead.	377
	Regular information events for family and legal representatives	473
Interdisciplinary collaboration	Shared conversation with legal representatives, trusted doctor and family	647
	Interdisciplinary team, that is responsible for covering all essential topics	571
	Support from trained staff	321
Awareness and autonomy	People who have a legal representative still know what they want for themselves and what they don’t want. Just because someone does not have a verbal language doesn’t mean they can’t speak for themselves.	363
	Perceiving people with disabilities as people whose understanding of certain topics can be huge if you manage to speak the right language.	359
	Seeing the client as autonomous persons with wishes and rights	270

### Barriers and facilitators for ACP

**Fig. 2:**
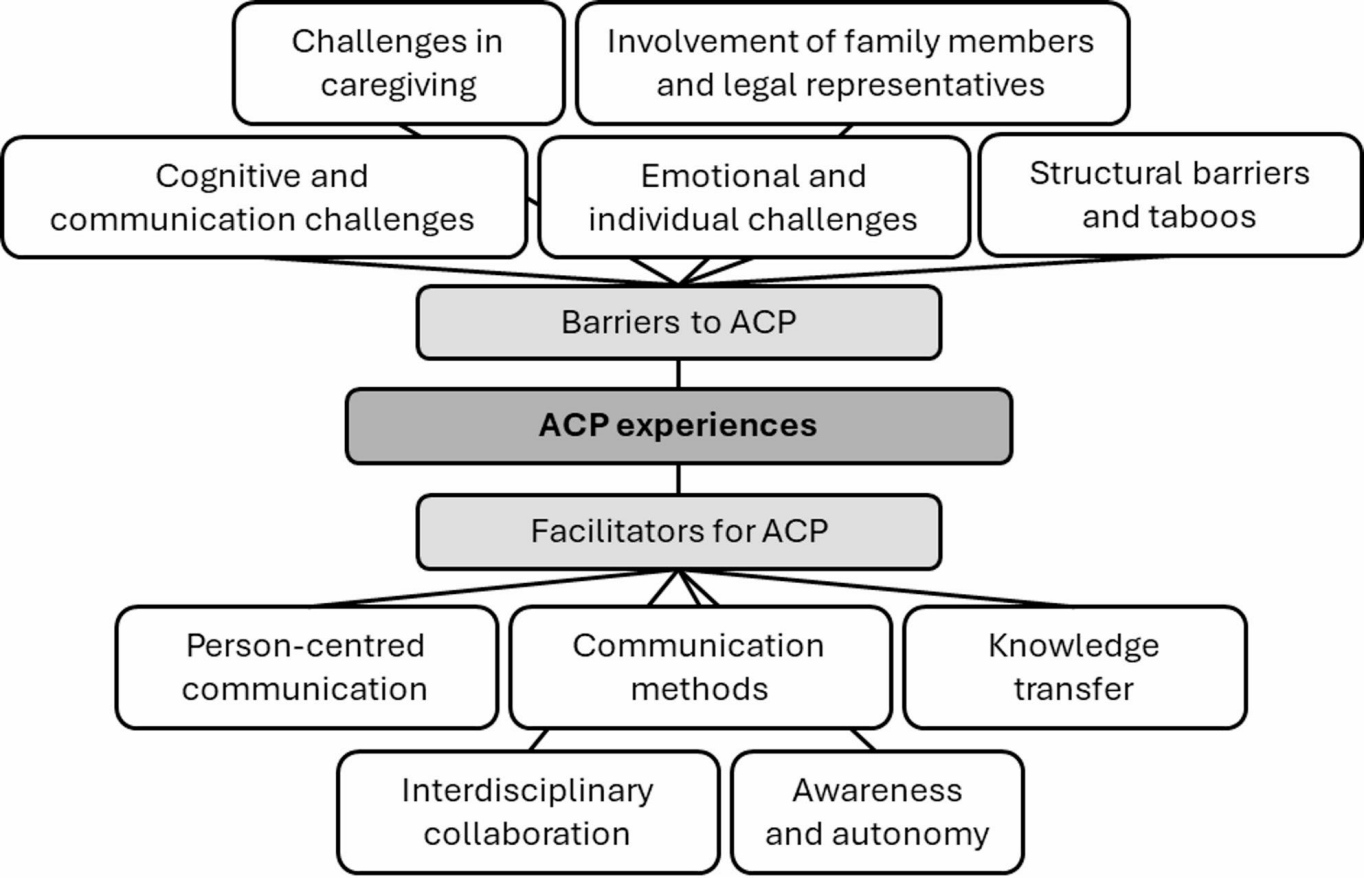
Barriers and facilitators to Advance Care Planning (ACP)

Caregivers were asked open-ended questions to identify barriers and facilitators for ACP with people with ID. They could give multiple reasons. A total of 106 caregivers indicated barriers to ACP, and 103 provided information about its facilitators. Five categories were identified as barriers to ACP (see Fig. 2; Table 3). The first category related to *Cognitive and Communication Challenges*. Many caregivers emphasized difficulties in assessing clients’ capacity to understand and participate in ACP. Clients were described as non-verbal or as having substantial difficulties in expressing themselves and understanding abstract or complex topics such as illness progression, death, or future planning. Caregivers reported difficulty in adapting their language to a suitable level and expressed uncertainty about the extent to which clients comprehended the content of the conversations. Limited attention spans and challenges in understanding temporal concepts were further cited as barriers.

The second category comprised *Emotional and Individual Challenges*. Emotional resistance was reported on both sides. Caregivers expressed concerns about causing anxiety or distress by initiating conversations about serious health issues. Clients were described as experiencing fear related to aging, dying, and medical interventions. Some caregivers reported a perceived general lack of interest or willingness among clients to engage with the topic.

The third category comprised *Structural Barriers and Taboos*. A lack of time, institutional support, and appropriate communication tools were identified as significant structural constraints. Caregivers reported insufficient time to conduct ACP. In many settings, aging and dying remained taboo topics and were not integrated into daily routines. A shortage of trained professionals and interdisciplinary collaboration was also noted.

The fourth category depicted *Challenges in Caregiving*. Some caregivers reported feeling inadequately prepared to discuss medical issues and unsure of whom to consult for support. Establishing trust with clients was considered essential but difficult, especially in the context of high staff turnover. Caregivers also highlighted difficulties in interpreting client preferences and in translating complex health scenarios into simplified, meaningful content.

The fifth category described the *Involvement of Family Members and Legal Representatives*. Professional caregivers described family members and legal guardians as potential sources of conflict or delay in the ACP process. Common issues cited included differences in views between clients and their representatives, emotional avoidance and a general lack of awareness regarding ACP. Some caregivers also perceived a limited willingness among relatives to engage with the topic or support clients’ autonomy.

Five categories emerged from the analysis of facilitators of ACP (see Fig. 2; Table 3). The first category was related to *Person-centred Communication*. Caregivers emphasized the importance of thorough preparation and creating a safe, empathetic, and trustful environment for ACP discussions. Strategies included conducting multiple, shorter sessions rather than a single lengthy one, allowing clients to pause or delay discussions when overwhelmed, and using relatable personal examples to foster understanding. Continuity in caregiving staff and exchange of personal experiences among peers were also cited as beneficial.

The second category comprised *Communication Methods*. The use of augmentative and alternative communication was highlighted as a crucial support. Suggestions included visual aids, easy-to-read materials, pictograms, and accessible brochures and websites, all tailored to the diverse needs of people with ID.

*Knowledge Transfer* was the third category. Caregivers identified the need for training, workshops, and professional development related to ACP. This education should extend beyond caregivers to include physicians, institutional staff, legal guardians, and family members. Proposals included establishing specialized training tracks and increasing institutional awareness and integration of ACP.

The fourth category was named *Interdisciplinary Collaboration*. The importance of cooperation across professional roles – such as general practitioners, specialists, nurses, educators, legal representatives, and home care providers – was underscored. Effective interdisciplinary teamwork was seen as essential for successful ACP implementation.

The fifth category was related to *Awareness and Autonomy*. Caregivers advocated for societal recognition of the rights and capabilities of individuals with ID. They stressed fostering autonomy through person-centred support and reducing substitute decision-making. The ability of non-verbal individuals to express preferences was acknowledged, with a call to normalize conversations about death to reduce associated fears.

## Discussion

The present study provides valuable insights into the current state of ACP usage for individuals with ID in Austria, highlighting both persistent barriers and promising facilitators. Despite the well-documented benefits of ACP, including improved quality of end-of-life care, increased satisfaction among professional caregivers and families, and enhanced patient autonomy, our findings reveal that ACP remains rarely practiced and poorly integrated into the care of people with ID.

Only one-third of professional caregivers reported having engaged in ACPdiscussions with their reference client, and the rates of documented advance directives and health care proxies were exceptionally low. Notably, a large proportion of caregivers were uncertain whether such documentation even existed for their client. This lack of awareness is particularly striking given that these respondents were the primary caregivers responsible for the client’s health-related matters, precisely the people who should be most informed about such planning [20].

Surprisingly, ACP engagement was statistically not associated with the client’s age or presence of a chronic illness. This is notable given that both older age and chronic health conditions are typically strong indicators for initiating ACP in the general population [35]. The absence of such an association in our sample suggests that ACP is not being used in a targeted or needs-based manner. Instead, engagement was shaped by relational and contextual factors. As shown in Table 3, caregivers often initiated ACP when prompted by factors in the client’s environment, such as family wishes or recent health events, rather than by clinical indicators. This pattern is consistent with previous research [17, 21, 23]. Some respondents also described ACP as arising from individual caregiver initiative, reflecting personal commitment and proactive, person-centred attitudes, which are known facilitators of ACP in both ID and general care settings [17, 19, 36]. Studies also highlight that ACP is rarely embedded in routine practice and often relies on individual initiative rather than health needs [17], and that role ambiguity and reluctance to start conversations further limit targeted ACP use [36, 37]. Together, these findings suggest that ecosystems of support, rather than client characteristics alone, frequently enable ACP engagement. This underscores the need for clear guidelines and standardized triggers for initiating ACP with people with ID. One reason for not engaging in ACP was the perceived inability of people with ID to understand or participate in such discussions, echoing findings from prior research that show widespread underestimation of the decision-making abilities of people with ID [1, 19]. While some people with severe and profound ID may indeed require significant support to engage in complex decision-making, the blanket assumption of incapacity is problematic. Such assumptions of incapacity extend well beyond ACP. Studies show that people with ID routinely encounter doubts about their decision-making ability in everyday matters, which restricts their autonomy and reinforces stigma [7, 38, 39]. This not only contravenes the principles of the UN-CRPD [31], which advocates for supported rather than substituted decision-making, but also reinforces a cycle of exclusion and silence around end-of-life issues. However, many individuals with ID want and are able to express their preferences and make informed decisions with appropriate support [22, 40–42]. This requires providing them with communication aids and tailored decision-making support to uphold their agency.

Emotional discomfort and avoidance, both among clients and caregivers, also emerged as key barriers. Some caregivers reported hesitancy to broach the subject for fear of causing distress, while others noted that clients themselves were reluctant to discuss future care. This is not unique to people with ID. A study on ACP in the general population revealed that conversations are hindered by people’s own emotions and fear of upsetting others [43]. However, in the ID-population, such conversations may be further complicated by prior lack of exposure to these topics or communicative limitations [19]. Supporting ACP in this context requires creating a safe, trusting environment and using person-centred approaches that allow conversations to unfold gradually and with sensitivity [25].

Organizational and structural challenges were also evident. Caregivers cited time constraints, the absence of clear institutional protocols, and lack of training as significant barriers. These issues mirror those reported in other studies and highlight the need for systemic change [20, 28]. Moreover, ACP was rarely embedded into standard procedures; only a few caregivers reported that discussions were initiated as part of institutional protocols. Described environments where conversations about aging and death remain taboo, or where staff turnover is high, establishing and maintaining consistent ACP practices can be particularly difficult, largely due to a lack of trust and rapport. Persistent cultural taboos surrounding end-of-life discussions, together with misconceptions about ID, stigma, and organizational shortcomings, continue to pose substantial barriers in Austria [37, 38]. However, the presence of such barriers also points toward concrete opportunities for intervention, especially at the organizational and policy levels.

Encouragingly, caregivers proposed several concrete strategies to help overcome the barriers they encounter in implementing ACP. Central to these suggestions was the emphasis on person-centred communication, adapting information to individual needs through accessible language, visual aids, and alternative communication methods. Rather than relying on isolated, one-time conversations, caregivers highlighted the importance of empathy, preparation, and sustained dialogue as the foundation for meaningful planning processes. They also underscored the value of interdisciplinary collaboration and targeted education; not only for themselves, but also for families, legal guardians, and medical professionals involved in care. These priorities reflect findings from previous research, which similarly identified effective communication, staff training, and collaborative approaches as critical enablers of successful ACP for people with and without ID [25, 28, 36, 44].

Another challenge identified in the data was the ambiguity surrounding who should initiate ACP. Some professional caregivers deferred responsibility to families, particularly in the context of legal guardianship. While family involvement is undoubtedly important, assuming that families alone should navigate or control ACP can result in delayed or absent planning. This further emphasises the importance of establishing clear roles, effective communication and formal protocols within care settings to enable better ACP discussions [37].

Our findings support a broader shift in thinking about autonomy and capacity. Even non-verbal people with ID or people with severe or profound ID are capable of expressing preferences when given appropriate support. Consistent with prior research, active support and needs-supportive environments have positive impacts on engagement, decision-making, and self-determination. This reinforces that with the right support, people with severe and profound ID can participate and make choices [41, 42]. Training that equips caregivers to recognize and respond to these expressions can help affirm the agency of people with ID and support their inclusion in planning for the future.

### Limitations, strengths and future directions

This study has several limitations that should be considered when interpreting the findings. First, the use of a convenience sampling strategy may have introduced selection bias, as caregivers who chose to participate might already have a particular interest in ACP or end-of-life topics, which may limit representativeness. Secondly, the survey did not collect information on the care setting (e.g. community-based living, supported accommodation or institutional care) or how often and for how long caregivers and clients interacted. These contextual factors likely influence opportunities, responsibilities, and expectations around ACP engagement, and future research should more precisely address these aspects. Third, we did not gather information on clients’ type or severity of ID, communication abilities, or additional support needs. Such characteristics may shape both opportunities and challenges for ACP and should be incorporated in future studies to allow more nuanced and stratified analyses. Finally, since the study was conducted in Austria, the findings may not be fully generalizable to other countries due to differences in legal frameworks, healthcare systems, and cultural attitudes toward disability and end-of-life care.

This study also has several strengths. It provides one of the first systematic examinations of ACP practices for people with ID in Austria, addressing an important evidence gap. The mixed-methods design allowed for integration of quantitative patterns with qualitative depth, giving insight into both the prevalence of ACP engagement and the real-world experiences of professional caregivers. The diversity of professional backgrounds in the sample strengthens the relevance of the findings across care roles. Importantly, the strong interest in ACP training among caregivers indicates a high readiness for practice improvement and supports the relevance of system-level interventions.

Future research should include people with ID as active participants and follow a participatory design (e.g., [45, 46]). Furthermore, studies should engage a broader range of stakeholders and explore ACP practices across different care settings and cultural contexts. Longitudinal or intervention-based studies are required in order to evaluate the impact of caregiver training, institutional support and policy frameworks on the implementation of ACP over time. It would also be valuable to examine how inclusive communication methods and decision-making supports influence the quality, accessibility, and outcomes of ACP discussions in everyday care.

## Conclusions

ACP remains the exception rather than the norm for people with ID in Austria. Despite its potential to improve person-centred, value-driven care, our findings highlight persistent gaps in awareness, training, and implementation. Caregivers often lack the tools, institutional frameworks, and confidence to initiate these essential conversations, and structural barriers continue to limit equitable access to ACP for people with ID. However, the strong interest in training and the identification of clear facilitators indicate a readiness for improvement.

Promoting equitable access to ACP requires systemic change, not just individual efforts. This includes integrating ACP into routine institutional practice, ensuring the availability of accessible communication tools, and training professional caregivers, families, and healthcare professionals to support inclusive decision-making processes. In line with the principles of the UN-CRPD, it is crucial to ensure that people with ID are meaningfully involved in decisions that affect their care and end-of-life experiences. Addressing these challenges is not only a matter of clinical best practice, but also of ethical, legal, and health equity responsibilities.

## Supplementary Information

Below is the link to the electronic supplementary material.

Supplementary Material 1

## Data Availability

The dataset generated and analysed during the current study is available in the OSF repository: 10.17605/OSF.IO/FEVPD.

## Abbreviations

ACP: Advance Care Planning
ID: Intellectual Disabilities
UN-CRPD: United Nations Convention on the Rights of Persons with Disabilities
SD: Standard Deviation
OSF: Open Science Framework

## References

[1] Voss H, Loxton A, Anderson J, Watson J. It was one of those complicated cases: health practitioners’ perspectives and practices of providing end-of-life care for people with profound intellectual and multiple disability. BMC Palliat Care. 2021;20(1):177. 34772382 10.1186/s12904-021-00873-5 PMC8586595

[2] Schalock RL, Luckasson R, Tassé MJ. An overview of *Intellectual disability: Definition, Diagnosis, Classification, and systems of supports*. Am J Intellect Dev Disabil. 2021;126(6):439–42. 12th ed. 34700345 10.1352/1944-7558-126.6.439

[3] Cooper SA, McLean G, Guthrie B, McConnachie A, Mercer S, Sullivan F, et al. Multiple physical and mental health comorbidity in adults with intellectual disabilities: population-based cross-sectional analysis. BMC Fam Pract. 2015;16(1):110. 26310664 10.1186/s12875-015-0329-3 PMC4551707

[4] Emerson E, Glover G, Hatton C, Wolstenholme J. Trends in age-standardised mortality rates and life expectancy of people with learning disabilities in Sheffield over a 33-year period. Tizard Learn Disabil Rev. 2014;19(2):90–5.

[5] Liao P, Vajdic C, Trollor J, Reppermund S. Prevalence and incidence of physical health conditions in people with intellectual disability – a systematic review. PLoS ONE. 2021;16(8):e0256294. 34428249 10.1371/journal.pone.0256294 PMC8384165

[6] Matin BK, Williamson HJ, Karyani AK, Rezaei S, Soofi M, Soltani S. Barriers in access to healthcare for women with disabilities: a systematic review in qualitative studies. BMC Women’s Health. 2021;21(1):44. 33516225 10.1186/s12905-021-01189-5 PMC7847569

[7] Ali A, Scior K, Ratti V, Strydom A, King M, Hassiotis A. Discrimination and other barriers to accessing health care: perspectives of patients with mild and moderate intellectual disability and their carers. PLoS ONE. 2013;8(8):e70855. 23951026 10.1371/journal.pone.0070855 PMC3741324

[8] Breuer MEJ, Bakker-van Gijssel EJ, Vlot-van Anrooij K, Tobi H, Leusink GL, Naaldenberg J. Exploring views on medical care for people with intellectual disabilities: an international concept mapping study. Int J Equity Health. 2022 July 19;21(1):99. 10.1186/s12939-022-01700-w PMC9295354 35854317

[9] Sítima G, Galhardo-Branco C, Reis-Pina P. Equity of access to palliative care: a scoping review. Int J Equity Health. 2024;23(1):248. 39581966 10.1186/s12939-024-02321-1 PMC11587758

[10] Adamidis F, Baumgartner NS, Kitta A, Kum L, Ecker F, Bär J, et al. Timely integration of palliative care: the reality check. A retrospective analysis. Support Care Cancer. 2024 Jul 17;32(8):518. 10.1007/s00520-024-08721-x PMC11254969 39017732

[11] Rietjens JAC, Sudore RL, Connolly M, van Delden JJ, Drickamer MA, Droger M, et al. Definition and recommendations for advance care planning: an international consensus supported by the European Association for Palliative Care. Lancet Oncol. 2017 Sept 1;18(9):e543–51. 10.1016/S1470-2045(17)30582-X 28884703

[12] Malhotra C, Shafiq M, Batcagan-Abueg APM. What is the evidence for efficacy of advance care planning in improving patient outcomes? A systematic review of randomised controlled trials. BMJ Open. 2022 July;12(7):e060201.

[13] Sudore RL, Heyland DK, Lum HD, Rietjens JAC, Korfage IJ, Ritchie CS, et al. Outcomes that define successful advance care planning: A Delphi panel consensus. J Pain Symptom Manag. 2018;55(2):245–e2558. 10.1016/j.jpainsymman.2017.08.025 PMC5794507 28865870

[14] van der Padt-Pruijsten A, Oostergo T, Leys MBL, van der Rijt CCD, van der Heide A. Hospitalisations of patients with cancer in the last stage of life. Reason to improve advance care planning? Eur J Cancer Care. 2022;31(6):e13720. 10.1111/ecc.13720 PMC9788226 36172990

[15] Brinkman-Stoppelenburg A, Rietjens JA, van der Heide A. The effects of advance care planning on end-of-life care: a systematic review. Palliat Med. 2014 Sept 1;28(8):1000–25. 10.1177/0269216314526272 24651708

[16] McMahan RD, Tellez I, Sudore RL. Deconstructing the complexities of advance care planning outcomes: what do we know and where do we go? A scoping review. J Am Geriatr Soc. 2021;69(1):234–44. 32894787 10.1111/jgs.16801 PMC7856112

[17] Voss H, Vogel A, Wagemans AMA, Francke AL, Metsemakers JFM, Courtens AM, et al. Advance care planning in palliative care for people with intellectual disabilities: A systematic review. J Pain Symptom Manage. 2017;54(6):938–e9601. 28797850 10.1016/j.jpainsymman.2017.04.016

[18] Adam E, Sleeman KE, Brearley S, Hunt K, Tuffrey-Wijne I. The palliative care needs of adults with intellectual disabilities and their access to palliative care services: a systematic review. Palliat Med. 2020 Sept;34(8):1006–18. 10.1177/0269216320932774 PMC7596767 32552409

[19] Foo B, Wiese M, Curryer B, Stancliffe RJ, Wilson NJ, Clayton JM. Specialist palliative care staff’s varying experiences of talking with people with intellectual disability about their dying and death: A thematic analysis of in-depth interviews. Palliat Med. 2021;35(4):738–49. 33730929 10.1177/0269216321998207

[20] Beck ER, McIlfatrick S, Hasson F, Leavey G. Nursing home manager’s knowledge, attitudes and beliefs about advance care planning for people with dementia in long-term care settings: a cross-sectional survey. J Clin Nurs. 2017;26(17–18):2633–45. 27995678 10.1111/jocn.13690

[21] Tuffrey-Wijne I, Finlayson J, Bernal J, Taggart L, Lam CKK, Todd S. Communicating about death and dying with adults with intellectual disabilities who are terminally ill or bereaved: A UK-wide survey of intellectual disability support staff. J Appl Res Intellect Disabil. 2020;33(5):927–38. 32072726 10.1111/jar.12714

[22] Tuffrey-Wijne I, Bernal J, Butler G, Hollins S, Curfs L. Using nominal group technique to investigate the views of people with intellectual disabilities on end-of-life care provision. J Adv Nurs. 2007;58(1):80–9. 17394619 10.1111/j.1365-2648.2007.04227.x

[23] Voss H, Vogel A, Wagemans AMA, Francke AL, Metsemakers JFM, Courtens AM, et al. What is important for advance care planning in the palliative phase of people with intellectual disabilities? A multi-perspective interview study. J Appl Res Intellect Disabil. 2020;33(2):160–71. 31441581 10.1111/jar.12653

[24] Cithambarm K, Duffy M, Courtney E. What constitutes good quality End-of-Life care? Perspectives of people with intellectual disabilities and their families. J Policy Pract Intellect Disabil. 2021;18(3):207–16.

[25] McKenzie N, Mirfin-Veitch B, Conder J, Brandford S. I’m still here: exploring what matters to people with intellectual disability during advance care planning. J Appl Res Intellect Disabil. 2017;30(6):1089–98. 28378405 10.1111/jar.12355

[26] Blaise M, Schroeder M, Suhrcke M, Komenda-Schned S, Zeilinger EL, Weber G. Exploring self-determination and satisfaction with life and services of people with disabilities. Int J Dev Disabil. 2025;early view:1–11.

[27] Bruun A, Cresswell A, Jordan L, Keagan-Bull R, Giles J, Gibson SL, et al. What are we planning, exactly? The perspectives of people with intellectual disabilities, their carers and professionals on end-of-life care planning: a focus group study. Palliat Med. 2024 June 1;38(6):669–78. 10.1177/02692163241250218 PMC11157974 38842172

[28] Voss H, Vogel AGFM, Wagemans AMA, Francke AL, Metsemakers JFM, Courtens AM, et al. Development, Implementation, and evaluation of an advance care planning program for professionals in palliative care of people with intellectual disability. Intellect Dev Disabil. 2021;59(1):39–54. 33543280 10.1352/1934-9556-59.1.39

[29] Gruber AS. Advance Care Planning in Österreich - Eine explorative Pilotstudie zur Testung des WEEA-Fragebogens [Internet]. Wien: educa Verlag; 2021 [cited 2022 Nov 6]. Available from: https://www.lehmanns.de/shop/sachbuch-ratgeber/56851108-9783903218253-advance-care-planning-in-oesterreich.

[30] McNamara B, Same A, Rosenwax L. Creating person-centred support for people with intellectual disabilities at the end of life: an Australian qualitative study of unmet needs and strategies. J Intellect Disabil. 2020;24(4):543–58. 30727802 10.1177/1744629518823887

[31] United Nations. Convention on the Rights of Persons with Disabilities (CRPD) [Internet]. 2006 [cited 2025 Jan 13]. Available from: https://social.desa.un.org/issues/disability/crpd/convention-on-the-rights-of-persons-with-disabilities-crpd#Fulltext.

[32] Sharma A, Minh Duc NT, Luu Lam Thang T, Nam NH, Ng SJ, Abbas KS, et al. A Consensus-Based checklist for reporting of survey studies (CROSS). J GEN INTERN MED. 2021;36(10):3179–87. 33886027 10.1007/s11606-021-06737-1 PMC8481359

[33] Leiner DJ. SoSci Survey (Version 3.4.22) [Computer software] [Internet]. 2023. Available from: https://www.soscisurvey.de.

[34] Elo S, Kyngäs H. The qualitative content analysis process. J Adv Nurs. 2008;62(1):107–15. 18352969 10.1111/j.1365-2648.2007.04569.x

[35] Mohan D, Sacks OA, O’Malley J, Rudolph M, Bynum J, Murphy M, et al. A new standard for advance care planning (ACP) conversations in the hospital: results from a Delphi panel. J GEN INTERN MED. 2021;36(1):69–76. 32816240 10.1007/s11606-020-06150-0 PMC7859119

[36] Vanderhaeghen B, Van Beek K, De Pril M, Bossuyt I, Menten J, Rober P. What do hospitalists experience as barriers and helpful factors for having ACP conversations? A systematic qualitative evidence synthesis. Perspect Public Health. 2019;139(2):97–105. 30010486 10.1177/1757913918786524

[37] Suen MHP, Chow AYM, Woo RKW, Yuen SK. What makes advance care planning discussion so difficult? A systematic review of the factors of advance care planning in healthcare settings. Palliat Support Care. 2024;22(6):2166–79. 10.1017/S1478951524000464 38766704

[38] Zeilinger EL, Stiehl KAM, Bagnall H, Scior K. Intellectual disability literacy and its connection to stigma: A multinational comparison study in three European countries. PLoS ONE. 2020;15(10):e0239936. 33057379 10.1371/journal.pone.0239936 PMC7561148

[39] Pelleboer-Gunnink HA, van Oorsouw WMWJ, van Weeghel J, Embregts PJCM. Stigma research in the field of intellectual disabilities: a scoping review on the perspective of care providers. Int J Dev Disabil. 2021;67(3):168–87. 10.1080/20473869.2019.1616990 PMC8211133 34188898

[40] Kirkendall A, Linton K, Farris S. Intellectual disabilities and decision making at end of life: A literature review. J Appl Res Intellect Disabil. 2017;30(6):982–94. 27456315 10.1111/jar.12270

[41] Beadle-Brown J, Beecham J, Leigh J, Whelton R, Richardson L. Outcomes and costs of skilled support for people with severe or profound intellectual disability and complex needs. J Appl Res Intellect Disabil. 2021;34(1):42–54. 32755061 10.1111/jar.12782

[42] Kuld PB, Frielink N, Zijlmans M, Schuengel C, Embregts PJCM. Promoting self-determination of persons with severe or profound intellectual disabilities: a systematic review and meta-analysis. J Intellect Disabil Res. 2023;67(7):589–629. 37165964 10.1111/jir.13036

[43] Graham-Wisener L, Nelson A, Byrne A, Islam I, Harrison C, Geddis J, et al. Understanding public attitudes to death talk and advance care planning in Northern Ireland using health behaviour change theory: a qualitative study. BMC Public Health. 2022;22:906. 35524295 10.1186/s12889-022-13319-1 PMC9077935

[44] Wichmann AB, van Dam H, Thoonsen B, Boer TA, Engels Y, Groenewoud AS. Advance care planning conversations with palliative patients: looking through the gp’s eyes. BMC Fam Pract. 2018;19(1):184. 30486774 10.1186/s12875-018-0868-5 PMC6263059

[45] Komenda-Schned S, Landskron SJ, Moritz P, Braunstein N, Hochmeister J, Riegler K, et al. Conceptualising good mental health for people with intellectual disabilities: an inclusive Delphi study. Int J Clin Health Psychol. 2025 July 1;25(3):100601. 10.1016/j.ijchp.2025.100601 PMC12269831 40678190

[46] Komenda-Schned S, Landskron SJ, Moritz P, Braunstein N, Hochmeister J, Riegler K, et al. Good mental health for people with intellectual disabilities: a participatory focus group study. Int J Equity Health. 2025 June 18;24(1):180. 10.1186/s12939-025-02562-8 PMC12175403 40533785

