# Supplementary material for: Barriers and facilitators to advance care planning for people with intellectual disabilities: a cross-sectional survey study of professional caregiver perspectives
Source: Int J Equity Health. 2025 Dec 27;25:29. doi: 10.1186/s12939-025-02747-1 (PMC12853855; doi:10.1186/s12939-025-02747-1)
Supplement: Supplementary file 1 — Supplementary Material 1 [file 12939_2025_2747_MOESM1_ESM.pdf]

Supplementary Material: German-language questionnaire

This questionnaire displays the content of the questions used for this study.

The survey was conducted online.

**SOZIODEMOGRAPHIE**							
**1. Wie alt sind Sie?**						Ich bin ____ Jahre alt	Keine Angaben
**2. Welchem Geschlecht fühlen Sie sich zugehörig?**							
	weiblich	männlich	divers	inter	offen	Keines der genannten	Keine Angaben
**3. Bitte geben Sie im Folgenden Ihre höchste abgeschlossene Ausbildung an:**							
Pflichtschule (Volksschule/ Grundschule)	Berufs-schule (Lehre)	Mittlere Schule/ Realschul-abschluss (ohne Matura/ Abitur)	Berufs-bildende/ Allgemein bildende Höhere Schule (Mit Matura/ Abitur)		Fachhoch-schule/ Universität	Anderes: ______	Keine Angabe

**4. Wie lange arbeiten Sie schon im Bereich Menschen mit Behinderungen?**						Seit _____ Jahren	Keine Angabe
Welche der folgenden Berufsbezeichnungen/Ausbildungen trifft am besten auf Sie zu [Mehrfach-Nennungen möglich]:							
Fachsozial-betreuer:in (Fachschule)	Pädagog:in (Heilpädagog:in, Sonder-pädagog:in)	Pflege-assistenz	Ergo-therapeut:in	Logopäd:in	Psycholog:in	Alltagsbe-gleiter:in	Anderes: ________

SCHULUNGEN / FORTBILDUNGEN			
**5.** Haben Sie schon einmal eine Fortbildung, Informationsveranstaltung, o.ä. zum Thema Vorausplanung der gesundheitlichen Versorgung/ Advance Care Planning besucht?	Ja	Nein	Keine Angabe
**6.** Haben Sie schon einmal eine Fortbildung, Informationsveranstaltung, o.ä. zum Thema Tod, Sterben, Trauer in Bezug zu Menschen mit IB besucht?	Ja	Nein	Keine Angabe
**7.** Hätten Sie Interesse an einer Fortbildung zum Thema Vorausplanung der gesundheitlichen Versorgung?	Ja	Nein	Keine Angabe
**8.** Hätten Sie Interesse an einer Fortbildung zum Thema Tod, Sterben, Trauer in Bezug zu Menschen mit IB?	Ja	Nein	Keine Angabe

NUTZUNG ACP BARRIEREN / HERAUSFORDERUNGEN / HILFREICHE FAKTOREN

Denken Sie an Ihre:n Bezugsklient:in.

Wenn Sie für mehrere Klient:innen die:der Bezugsbetreuer:in sind, denken Sie hier bitte an eine:n bestimmte:n Klient:in: Wählen Sie jene Person, für die Sie sich in gesundheitlichen Belangen am zuständigsten fühlen, und/oder die Sie besonders gut kennen.

**9.** Hat die Person eine schwere, chronische Erkrankung, z.B. Krebs, einen Herzfehler, eine Lungenerkrankung?				Ja	Nein	Weiß nicht	Keine Angabe
**10.** Wie alt ist die Person?						____ Jahre alt	Keine Angabe
**11.** Welchem Geschlecht fühlt sich die Person zugehörig?	weiblich	männlich	divers	inter	offen	Weiß ich nicht	Keine Angabe
**12.** Hat die Person eine Vorsorgevollmacht?				Ja	Nein	Weiß ich nicht	Keine Angabe
**13.** Hat die Person eine Patientenverfügung?				Ja	Nein	Weiß ich nicht	Keine Angabe
**14.** Wurden mit der Person schon einmal Gespräche zur Vorausplanung der gesundheitlichen Versorgung (ACP) geführt?					Ja	Nein	Keine Angabe

**Wenn 14 = ja:**<br>- **15. Falls möglich, führen Sie bitte Gründe an, warum es Gespräche diesbezüglich gab:**	Mögliche Gründe:<br>________________________

- **16. Welche Personen waren in die Gespräche involviert (kreuzen Sie alle zutreffenden an):**

	Person mit intellektueller Behinderung	Bezugs-betreuer:in	Erwachsenen -vertreter:in	Angehörige	Freunde	Andere ________

Wenn 14 = nein:

- **17. Falls möglich, führen Sie bitte Gründe an, warum es keine Gespräche diesbezüglich gab.**

18. Nennen Sie bitte 1-5 Herausforderungen, die Ihnen bei Gesprächen zur Vorausplanung der gesundheitlichen Versorgung mit Personen mit intellektueller Behinderung begegnet sind oder begegnen könnten:

☐ ___________________________
☐ ___________________________
☐ ___________________________
☐ ___________________________
☐ ___________________________

19. Nennen Sie bitte 1-5 Faktoren, die dabei helfen könnten, diese Herausforderungen oder Schwierigkeiten zu bewältigen:

☐ ___________________________
☐ ___________________________
☐ ___________________________
☐ ___________________________
☐ ___________________________

20. Abschluss: Offen Frage: Wollen Sie uns noch etwas mitteilen?: ______________________________

Nach Abschluss der Befragung werden den Teilnehmer:innen Links zu weiteren Informationen zum Thema Advanced Care Planning zur Verfügung gestellt.
